# Intravenous thrombolysis in acute central retinal artery occlusion – A prospective interventional case series

**DOI:** 10.1371/journal.pone.0198114

**Published:** 2018-05-29

**Authors:** Maximilian Schultheiss, Florian Härtig, Martin S. Spitzer, Nicolas Feltgen, Bernhard Spitzer, Johannes Hüsing, André Rupp, Ulf Ziemann, Karl U. Bartz-Schmidt, Sven Poli

**Affiliations:** 1 Department of Ophthalmology, University Medical Center Hamburg-Eppendorf (UKE), Hamburg, Germany; 2 University Eye Hospital Tübingen, University of Tübingen, Tübingen, Germany; 3 Department of Neurology & Stroke, and Hertie Institute for Clinical Brain Research, University Hospital Tübingen, Tübingen, Germany; 4 Department of Ophthalmology, University Medical Center Goettingen, Goettingen, Germany; 5 Department of Experimental Psychology, University of Oxford, Oxford, United Kingdom; 6 Coordination Center for Clinical Trials, University Hospital Heidelberg, Heidelberg, Germany; 7 Department of Neurology, University Hospital Heidelberg, Heidelberg, Germany; Fraunhofer Research Institution of Marine Biotechnology, GERMANY

## Abstract

**Background:**

No evidence-based therapy exists for non-arteritic central retinal artery occlusion (NA-CRAO). Retinal ischemic tolerance is low; irreversible damage occurs within four hours of experimental NA-CRAO. In previous randomized trials evaluating intra-arterial or intravenous thrombolysis (IVT) in NA-CRAO, only one patient was treated this early. In December 2013, the Departments of Neurology & Stroke and Ophthalmology at University Hospital Tuebingen, Germany, decided to treat patients using IVT within 4.5 hours of NA-CRAO, the therapeutic window established for ischemic stroke.

**Materials and methods:**

Consecutive NA-CRAO patients with severe visual loss received IVT after exclusion of intracranial hemorrhage. Follow-up was conducted at day 5 (d5) and day 30 (d30). Visual outcomes were compared to the conservative standard treatment (CST) arm of the EAGLE-trial.

**Results:**

Until August 2016, 20 patients received IVT within 4.5 hours after NA-CRAO with a median onset-to-treatment time of 210 minutes (IQR 120–240). Visual acuity improved from baseline mean logarithm of the minimum angle of resolution 2.46±0.33 (SD) (light perception) to 1.52±1.09 (Snellen equivalent: 6/200) at d5 (p = 0.002) and 1.60±1.08 (Snellen equivalent: 6/240) at d30. Compared to the EAGLE CST-arm, functional recovery to reading ability occurred more frequently after IVT: 6/20 (30%) versus 1/39 (3%) at d5 (p = 0.005) and at d30 5/20 (25%) versus 2/37 (5%) (p = 0.045). Two patients experienced serious adverse events (one angioedema and one bleeding from an abdominal aortic aneurysm) but recovered without sequelae.

**Conclusions:**

IVT within 4.5 hours after symptom onset may represent an effective treatment of NA-CRAO. Randomized trials are warranted to evaluate efficacy and safety of early IVT in acute NA-CRAO.

## Introduction

Acute central retinal artery occlusion (CRAO) leads to severe and permanent visual loss in the affected eye in more than 90% of cases [1]. Unfortunately, there is currently no effective treatment for CRAO endorsed by ophthalmological guidelines [2].

Thromboembolism is the prevailing mechanism in CRAO; only 5%, defined as arteritic CRAO, are associated with inflammatory changes [3].

Preclinical models of non-arteritic CRAO (NA-CRAO), suggest a limited ischemia tolerance of the retina with irreversible damage occurring within four hours after cessation of blood flow [4–5]. Consequently, in the absence of restorative treatments, early reperfusion must be considered the pivotal requirement for successful treatment. In acute ischemic stroke, for which similar pathophysiology and therapeutic time window may be assumed, intravenous thrombolysis (IVT) with tissue plasminogen activator (tPA) initiated within 4.5 hours after symptom onset increases rates of early reperfusion and good neurological outcome [6].

After the negative results of the randomized EAGLE-trial, intra-arterial thrombolysis (IAT) for NA-CRAO was widely abandoned [7]. A second randomized, placebo-controlled trial by Chen and colleagues investigating IVT in acute NA-CRAO, was prematurely terminated due to safety concerns after enrolment of just 16 patients and unable to show a significant IVT effect [8]. Enrolment windows in these trials, however, were 20 and 24 hours respectively. Only one patient in the IVT trial and no patient in the EAGLE-trial received thrombolytic treatment within 4.5 hours.

More extensive data on early IVT in NA-CRAO is only available from case series. Schrag and colleagues recently published a patient-level meta-analysis in which visual outcomes of 34 patients who received IVT within 4.5 hours are summarized [9]. Favorable outcomes, defined by a recovery of best corrected visual acuity (BCVA) to logarithm of the minimum angle of resolution (LogMAR) ≤0.7 (LogMAR 0 corresponds to normal vision (100%; Snellen equivalent: 6/6, higher values indicate poorer vision; e.g. LogMAR 0.7 corresponds to 20% of normal visual acuity; Snellen equivalent: 6/30), were reported in 50% of those patients and thus, significantly more often than in non-treated patients and patients receiving non evidence-based conservative standard treatment (CST) [2]. No significant effect was found for IVT beyond 4.5 hours.

In a later French retrospective multicenter analysis of 30 NA-CRAO patients, IVT within 6 hours (17/30 within 4.5 hours) lead to significant visual improvement in 55% of cases; rates of functional recovery were not reported [10]. Three patients suffered intracerebral hemorrhage, one of which was symptomatic. Nedelmann and colleagues identified the sonographically detected retrobulbar spot sign as a predictor of poor IVT-response [11]. Out of eleven patients, who received IVT within 12 hours of NA-CRAO onset, all spot sign-negative patients (4/4) but no (0/7) spot sign-positive patient experienced functional recovery. For the seven patients treated within 4.5 hours, functional recovery was seen in two (29%), which is in line with Schrag’s findings. No serious adverse events (SAE) related to IVT occurred.

These findings support the efficacy of early IVT in acute NA-CRAO. However, data was gathered in ten rather small prospective case series, with the two largest including only seven patients treated within 4.5 hours, and one larger retrospective analysis [10–12].

Furthermore, tPA–the drug established for IVT in ischemic stroke–was only used in the four most recent case series [10–13]. Consequently, neither efficacy nor safety of IVT in NA-CRAO can be regarded as established.

In order to add to the growing evidence of IVT in NA-CRAO, in December 2013, we established a standard operating procedure (SOP) at our institution for the emergency diagnosis and treatment of acute NA-CRAO including IVT with tPA initiated within 4.5 hours of symptom onset. Due to associated time loss and invasiveness, we decided against the use of IAT. We report on visual outcomes and safety and propose a sample size calculation for a multi-center prospective randomized placebo-controlled phase III trial.

## Materials and methods

### Study design

Single-center, prospective interventional case series with partially-blinded outcome assessment. Outcomes were compared to the EAGLE CST-arm. Institutional Review Board approval was obtained from the independent ethics committee of Tübingen University (protocol-no 564/2016BO2) and all clinical investigation have been conducted according to the principles expressed in the Declaration of Helsinki. Written informed consent was obtained from all patients prior to IVT.

### Setting, patient eligibility and treatment

The study was conducted at the Eye Hospital and the Department of Neurology & Stroke of Tübingen University Hospital, a tertiary care facility.

Diagnosis of NA-CRAO was confirmed by an experienced ophthalmologist independent of the study through assessment of BCVA using Early Treatment Diabetic Retinopathy Study (ETDRS) charts, intraocular pressure, relative afferent pupil defect, slit-lamp biomicroscopy and fundoscopy to exclude rare differential diagnoses associated with sudden, painless, and severe visual loss, e.g. retinal detachment or hemorrhage. To avoid delay of IVT, fluorescein angiography and assessment of visual fields were not performed prior to treatment.

For inclusion, BCVA had to be LogMAR ≥1.3 (Snellen equivalent: 6/120), corresponding to functional blindness according to the World Health Organization International Classification of Diseases (WHO-ICD) [14]. Categories of low vision (counting fingers, hand motion, light perception and no light perception) were assessed at a distance of 30 cm and translated into LogMAR values following suggestions from Lange [15].

In addition to ophthalmological assessment, all patients received a thorough neurological examination including blood sampling, National Institutes of Health Stroke Scale score (NIHSS) and a cranial computed tomography (CT) or magnetic resonance imaging (MRI) to exclude intracranial hemorrhage (ICH), (sub-acute) cerebral infarction, and other IVT contraindications (see supplemental Methods).

In analogy to acute ischemic stroke, eligible patients received 0.9 mg of tPA (Actilyse®, Boehringer Ingelheim, Germany) per kilogram of bodyweight (max. 90 mg, 10% as bolus and the remainder over one hour) within 4.5 hours of self-reported symptom onset.

All patients were admitted to the Stroke Unit of the Department of Neurology & Stroke. Brimonidine or dorzolamide/timolol was applied to normalize increased intraocular pressure in two cases. No further CST was administered.

### Contraindications for intravenous thrombolysis

Patients with acute non-arteritic central retinal occlusion were excluded from intravenous thrombolysis according to the official contraindications provided by the European Medicines Agency for use of tissue plasminogen activator within 4.5 hours of ischemic stroke onset [16]; exception was made for patients “over 80 years of age”, “with any history of prior stroke and concomitant diabetes”, and “on vitamin K antagonists and INR ≤1.7” (all ex- and inclusion criteria are listed in S1 Table).

### Ophthalmological follow-up

All patients received ophthalmological follow-up (FU) examinations at pre-specified time points, i.e. at day 5±2 after IVT or prior to discharge (whichever occurred first) (d5) and at day 30±5 (d30). BCVA was determined at each visit by an experienced ophthalmologist independent of the study. Functional recovery was pre-defined as a BCVA of LogMAR ≤0.5 (Snellen equivalent: 6/20) (reading ability according to the WHO-ICD [14]).

### Blinding

All ophthalmologists performing FU examinations were blinded to prior BCVA.

### Neurological follow-up

All patients underwent at least 24 hours of stroke unit monitoring. FU brain imaging (CT or MRI) was performed 24±12 hours after IVT as part of clinical routine and evaluated by board-certified neuroradiologists independent of the study for presence of ICH, new ischemic lesions, and subcortical leukoencephalopathy.

All patients underwent thorough etiological work-up including neurovascular ultrasound, 24-hour electrocardiographic monitoring, echocardiography and laboratory inflammatory markers for exclusion of arteritic CRAO.

NIHSS was assessed at d5. Modified Rankin Scale score (mRS) and the occurrence of SAE were assessed at d30.

### Comparison to conservative standard treatment

For estimation of IVT effect, we compared visual outcomes with individual patient data taken from the EAGLE CST-arm provided by the EAGLE Study Group (see original publication for patient baseline characteristics [7]). Of the 40 patients in the EAGLE CST-arm, 39 received FU at d5 and 37 at d5 and d30.

### Statistics

Statistical analysis was performed using SPSS Statistics 24 (IBM, USA). One-factorial repeated-measurements analysis of variance (ANOVA) was used to assess intra-individual differences from baseline to d5 and d30 in the respective treatment group. To analyze differences between groups, ANOVA with two independent factors (IVT and CST) and time was used. Significant values were corrected for inhomogeneous variance according to Greenhouse-Geisser. Student’s t-test was performed for dependent means; significant values were corrected for multiple testing according to Bonferroni. Fisher’s exact test was employed to determine differences in the proportions of functional recovery between study groups. P <0.05 was considered significant. Power calculation for a suggested future randomized trial was done using the function power.prop.test from R version 3.2.2 stats package (The R Foundation, Austria). Reporting in accordance with the TREND guidelines for non-randomized interventional trials.

## Results

### Patient population

**Fig 1:**
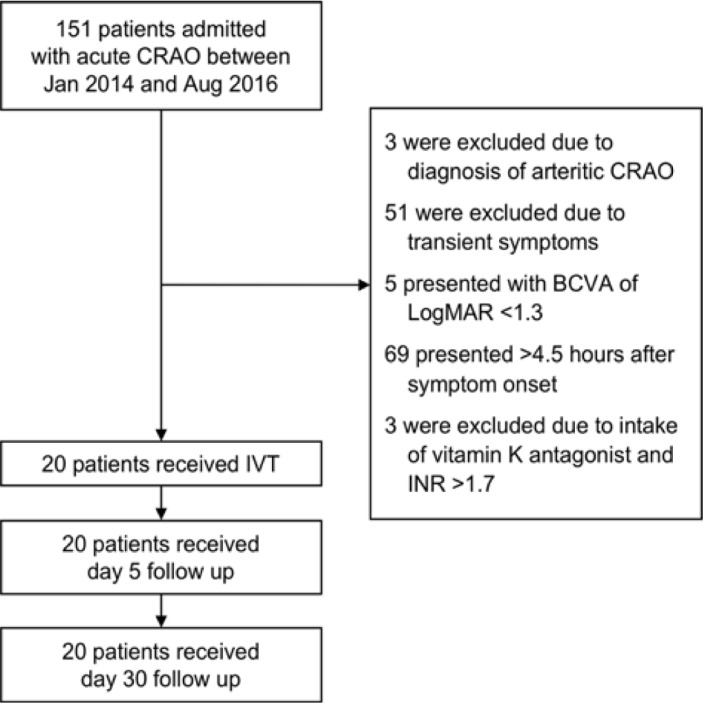
Patient flow. BCVA = best corrected visual acuity, CRAO = central retinal artery occlusion, IVT = intravenous thrombolysis, INR = international normalized ratio, LogMAR = logarithm of the minimum angle of resolution.

Between January 2014 and August 2016, 20 consecutive patients with acute NA-CRAO received IVT according to our SOP (see Table 1 for baseline characteristics, and S2 Table for individual patient data). Patient flow is indicated in Fig 1.

**Table 1:** Patient characteristics.

Sex, female^1^	10 (50%)
Age, years^2^	72.8±10.9
Affected eye, right^1^	13 (65%)
Historical best corrected visual acuity of affected eye, LogMAR^3^	0 (0–0)
Symptom onset to initiation of IVT, minutes^2^	183.5±62.0
**Neurological and neuroradiological assessment prior to IVT**	
Premorbid modified Rankin Scale score^3^	0 (0–0)
National Institutes of Health Stroke Scale score prior to IVT^3^	0 (0–0)
Systolic blood pressure prior to IVT, mmHg^2^	152.8±12.6
CT/MRI prior to IVT^1^	19 (95%)/1 (5%)
White matter changes: mild/moderate to severe	8 (40%)/6 (30%)
**Vascular risk factors**		
Arterial hypertension^1^	17 (85%)
Diabetes mellitus^1^	6 (30%)
Hyperlipidemia^1^	14 (70%)
Atrial fibrillation^1^	1 (5%)
Ischaemic heart disease or history of myocardial infarction^1^	4 (20%)
Congestive heart failure^1^	3 (15%)
Active smoking^1^	7 (35%)
History of stroke^1^	4 (20%)
**Prior antithrombotic treatment**		
Acetylsalicylic acid^1^	6 (30%)
Clopidogrel^1^	1 (5%)
Dual antiplatelet therapy^1^	1 (5%)
**Suspected etiology of non-arteritic central retinal artery occlusion**^**4**^		
Carotid artery stenosis^1^	3 (15%)
Atrial fibrillation^1^	1 (5%)
Cryptogenic^1^	16 (80%)

^1^number (%)

^2^mean±standard deviation

^3^median (interquartile range)

^4^according to the Trial of Org 10172 in Acute Stroke Treatment (TOAST) classification [17]; CT = computed tomography, IVT = intravenous thrombolysis, LogMAR = logarithm of the minimum angle of resolution, MRI = magnetic resonance imaging

### Treatment and visual outcome

**Fig 2:**
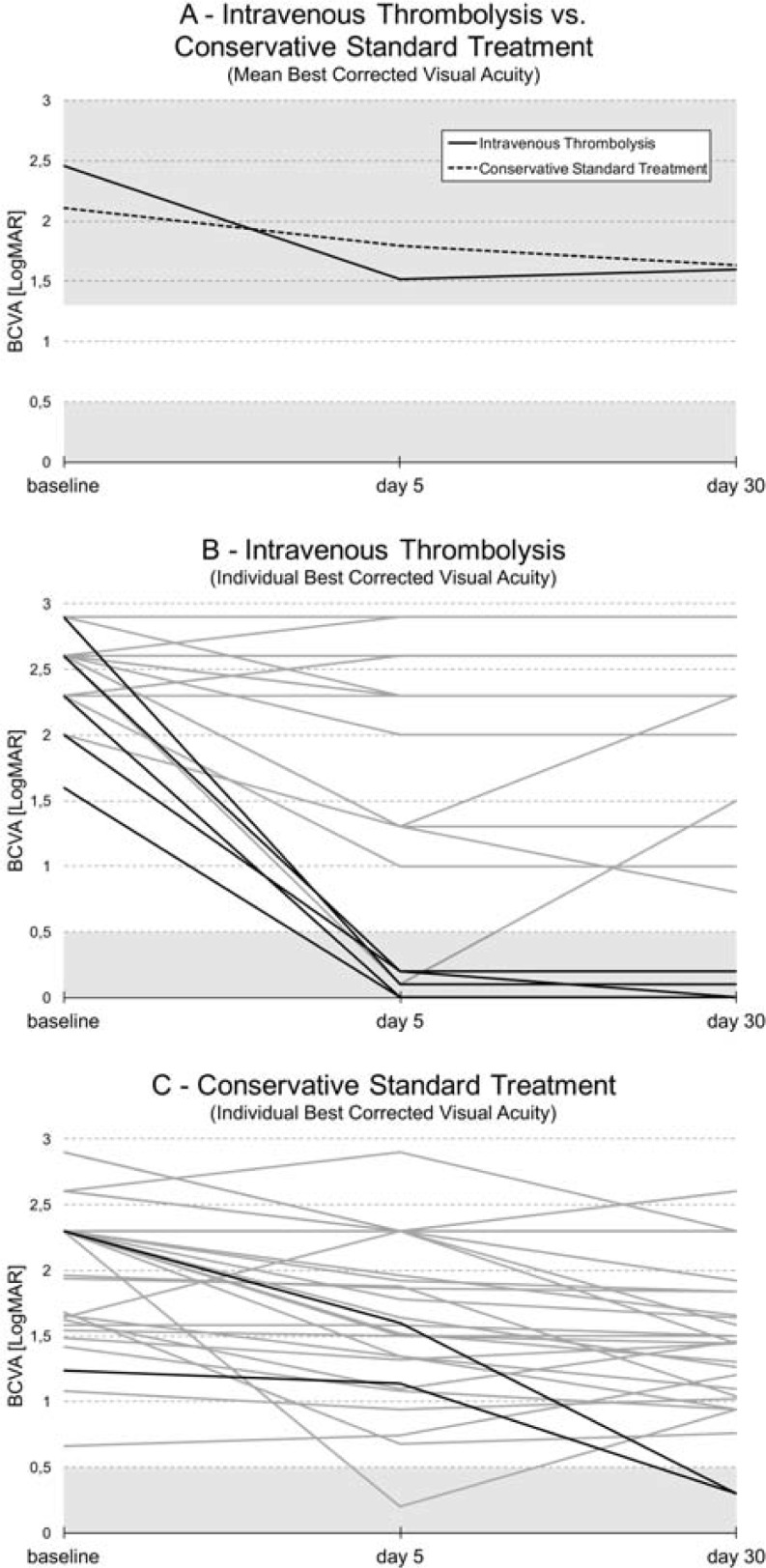
**Evolution of best corrected visual acuity over time (BCVA):** (A) mean BCVA of our intravenous thrombolysis (IVT) cohort (N = 20) and of the conservative standard treatment (CST) group of the EAGLE-trial [7]. (B) individual BCVA of our IVT-cohort and (C) of the EAGLE CST-arm. Functional blindness (LogMAR >1.3) and functional recovery (LogMAR ≤0.5) are indicated by a gray and blue background, respectively. LogMAR = logarithm of the minimum angle of resolution.

Median onset-to-treatment time was 210 minutes (interquartile range (IQR) 120–240). Mean BCVA was LogMAR 2.46±0.33 (standard deviation) (light perception) on admission and improved to 1.52±1.09 (Snellen equivalent: 6/200) at d5 (p = 0.002) and 1.60±1.08 (Snellen equivalent: 6/240) at d30 (vs. baseline p = 0.004, vs. d5 n.s.) (Fig 2A).

Ophthalmological FU examinations were performed beyond d30 in 14 patients. Median time between NA-CRAO and latest FU was 6 months (IQR 4–11.5). Mean BCVA at the latest FU was LogMAR 1.39±1.15 (Snellen equivalent: 6/150) and did not differ from BCVA at d30 (1.51±1.11, n = 14) (Snellen equivalent: 6/200).

Improvement of BCVA from baseline to FU was also observed in the EAGLE CST-arm (N = 37; baseline LogMAR 2.09±0.51 (counting fingers) vs. d5 LogMAR 1.78±0.60 (Snellen equivalent: 6/380), p = 0.001; baseline vs. d30 LogMAR 1.63±0.62 (Snellen equivalent: 6/240), p<0.001; d5 vs. d30, n.s.). However, the gain in BCVA from baseline to d30 was greater in our IVT-treated cohort (F(1.459, 110) = 6.601, p = 0.005; Fig 2A).

**Fig 3:**
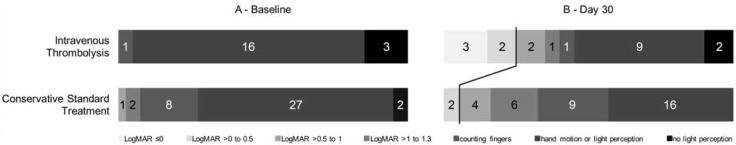
Categorical presentation of best corrected visual acuity. Categorical presentation of best corrected visual acuity (according to the current version of the WHO International Classification of Diseases [14]) at baseline and at day 30 of our intravenous thrombolysis cohort and of the conservative standard treatment group of the EAGLE-trial [7]. We defined favorable outcome as mild or no visual impairment (LogMAR ≤0.5, indicated in blue). Unfavorable outcome includes moderate or severe visual impairment (LogMAR >0.5 to ≤1.3) and functional blindness (LogMAR >1.3). LogMAR = logarithm of the minimum angle of resolution.

Despite mean baseline BCVA being worse in our group compared to the EAGLE CST-arm (p = 0.018; Fig 2A), the rate of functional recovery to LogMAR ≤0.5 (Snellen equivalent: 6/20) was higher: 6/20 (30%) vs. 1/39 (3%) at d5 (p = 0.005) and 5/20 (25%) vs. 2/37 (5%) at d30 (p = 0.045); compare Figs 2B, 2C and 3.

One patient in our cohort, who had recovered to LogMAR 0.0 (Snellen equivalent: 6/6) at d5, suffered a second NA-CRAO in the same eye two weeks later. IVT was not repeated due to multiple sub-clinical small acute and subacute cerebral infarcts seen on MRI. The patient’s final BCVA was LogMAR 1.5 (Snellen equivalent: 6/200) at d30.

Another patient, with a history of age-related macular degeneration and prior BCVA of LogMAR 1.0 (Snellen equivalent: 6/60) in the right eye and 2.3 (perception of hand motion) in the left eye, presented with an acute NA-CRAO of the right eye, which further reduced BCVA to LogMAR 2.3. It was decided to apply IVT in an attempt to avert even more severe visual impairment. At d5 and d30 FU, the patient had recovered to pre-NA-CRAO BCVA. For statistical analysis, we classified both patients as not recovered.

### Safety and neurological outcome

IVT-related SAE occurred in two patients. One patient suffered orolingual angioedema, which was treated with single-dose antihistamine and prednisolone and did not require invasive airway-management. Another patient suffered hemorrhage from an abdominal aortic aneurysm of which the attending neurologist was unaware at the time of IVT. The bleed led to a relevant drop in hemoglobin levels (from 4.97 to 3.35 mmol/L) and required a single transfusion of packed red blood cells. Both patients completely recovered from the respective SAE.

No intracranial or intraocular hemorrhage was observed. Silent cerebral infarcts were seen in 3/20 (15%).

Baseline NIHSS was 0 in all patients apart from two, who presented with an NIHSS of 1. One patient with known dementia scored 1 due to impaired orientation. The other patient reported mild sensory loss of the right face and arm, which had occurred simultaneously to NA-CRAO and resolved within 24 hours after IVT.

Median pre-NA-CRAO mRS was 0 (IQR 0–1). Median mRS at d30 was 2 (IQR 1.5–2) due to visual loss.

## Discussion

This single-center prospective interventional case series describes the visual and neurological outcome of 20 consecutive NA-CRAO patients treated with IVT within a 4.5-hour time window. The higher rate of functional recovery indicates that, in comparison to CST, early IVT with tPA could improve visual outcomes after NA-CRAO. Despite differences in study design and population characteristics including the underlying NA-CRAO causes, our results are in line with recently published data [9, 11]. The study of Nedelmann et al. [11] and our study were prospectively conducted at one respective center, whereas Schrag and colleagues [9] and Preterre et al. [10] published retrospectively collected data from multiple centers. The therapeutic time windows for IVT ranged from 4.5 up to 12 hours after symptom onset [9–11], and evaluation of visual acuity was performed either in a continuous [10–11] or a dichotomized manner [9]. Cryptogenic NA-CRAO seems overrepresented in our cohort (80%) especially compared to Nedelmann’s study in which 64% of NA-CRAO cases were attributed to large artery arteriosclerosis [11]. However, definitions differ between studies: in our cohort, 60% of NA-CRAO cases (12/20) would have been classified as due to large artery arteriosclerosis if using Nedelmann’s broader definition, i.e. severe arteriosclerosis of carotid arteries and aortic arch (see S2 Table). This is of major importance as clot’s nature and properties might significantly influence IVT efficacy. However, the extent as well as the time course of (permanent) ischemic retinal damage is defined by the time and extent of vessel occlusion (i.e. CRAO with or without residual retinal circulation *versus* cilioretinal artery sparing *versus* branch retinal artery occlusion) and not primarily by clot composition.

### Safety

We observed two IVT-related SAE; both required intervention but did not cause permanent damage. No intracranial or intraocular hemorrhage was observed in our IVT-cohort.

Overall, we would expect the rate of IVT-related adverse events in NA-CRAO to be equal to those seen in minor stroke (2 to 2.4% [18–19]) or even lower. Increased ICH rates as observed in the EAGLE- (5%) [7] and the Chen-trial (12.5%) [8] may be due to delay of thrombolytic treatment far beyond 4.5 hours after symptom onset. Previous IVT-trials in stroke excluded patients exceeding the time windows of 4.5 to 6 hours to avoid an assumed increase in ICH risk associated with progressive blood-brain barrier disruption. MRI studies have revealed concurrent subclinical cerebral infarction in up to 25% of NA-CRAO cases [20].

### Risk and benefit of IVT in NA-CRAO

Patients not recovering from NA-CRAO suffer sustained disability due to visual loss, reflected by an mRS of 2. This instance justifies aggressive treatment despite an increased risk for adverse events.

We agree with Schrag that only a randomized placebo-controlled double-blinded phase III trial may conclusively answer the question whether IVT initiated within 4.5 hours is efficacious and safe for NA-CRAO treatment.

### Important considerations for a clinical phase III trial

First and foremost, the choice of clinical endpoint is crucial to the success of such a trial. Analyses of changes in mean BCVA–recently presented in [10]–are not suited to evaluate IVT efficacy in NA-CRAO, as significant changes in LogMAR do not necessarily reflect clinically relevant improvement or deterioration: a change from LogMAR 2.5 to 1.5 (as observed in both our IVT-cohort and the EAGLE CST-arm) (Snellen equivalent: light perception to 6/200) basically represents no relevant improvement as both values correspond to functional blindness [14], whereas a change from 1.5 to 0.5 (Snellen equivalent: 6/200 to 6/20) signifies recovery from functional blindness to reading ability. We consequently suggest a dichotomized analysis of visual outcome data and the use of a clinically relevant primary efficacy endpoint, i.e. regain of reading ability (LogMAR ≥0.5) (Snellen equivalent: 6/20), to distinguish favorable from unfavorable outcome.

It will be necessary to limit enrolment to patients with previously healthy eyes (LogMAR ≤0.5; Snellen equivalent: 6/20) and severely impaired vision on admission (LogMAR ≥1.3; Snellen equivalent: 6/120), i.e. functional blindness according to WHO-ICD [14]). Normal vision prior to NA-CRAO is the precondition for reaching the chosen clinical endpoint of functional recovery and will avoid potential bias through unbalanced baseline BCVA. Additionally, only severe visual loss in a previously healthy eye with realistic chances of full recovery and relevant disability as an alternative outcome, justifies the use of a potentially harmful therapy. Furthermore, a sudden and painless steep drop in BCVA most reliably indicates the presence of NA-CRAO with a complete and proximal vessel occlusion and reduces the risk of including patients with other differential diagnoses to an absolute minimum.

To assess the feasibility of a phase III trial, we performed power-calculations with the clinical endpoint of functional recovery to reading ability at d30 after NA-CRAO.

Based on rates of functional recovery to LogMAR ≤0.5 (Snellen equivalent: 6/20) in our IVT-cohort (25%) and that of Nedelmann (29%) and Schrag (50% to LogMAR ≤0.7; Snellen equivalent: 6/30), we conservatively estimate the success rate in the intervention-arm to be 20%. Based on recovery rates in the EAGLE CST-arm (5.4%) and Schrag’s meta-analysis (7.4%) [9], we optimistically assume a success rate of 10% in the placebo-arm. A two-armed randomized trial with a 10% drop-out rate would require 442 patients in order to detect a treatment effect at the 0.05-level with a power of 80%, using Chi-squared approximation. Our enrolment rate indicates that such a trial could be performed within three years if at least 20 study centers are involved.

In our case series, the majority of NA-CRAO patients with relevant persisting visual loss was ineligible for treatment due to late admission (69/100). In analogy to IVT in ischemic stroke [21], educational efforts will have to be undertaken to decrease admission delays.

The use of prognostic biomarkers, like the previously discussed retrobulbar spot sign [11], may enrich a study population and reduce sample size. However, it may also complicate and slow down enrolment. Additionally, spot sign-positive patients would be left untreated whilst futility of IVT in these patients is not yet established. We therefore strongly recommend, that the evaluation of the retrobulbar spot sign and its prognostic value should be part of the proposed randomized trial, but current data does not support exclusion of patients on its basis.

### Strengths and limitations

Several limitations of our study require further discussion. It is a non-randomized prospective interventional case series performed at a single tertiary care center. However, no published data is available from randomized trials studying IVT (or IAT) within a 4.5-hour time window after NA-CRAO.

Including 20 consecutively treated patients, with none lost to FU, this case series is the largest prospective study of early IVT in NA-CRAO. Our outcome data is in line with previously published studies [9, 11].

The control group used for estimation of IVT efficacy was taken from the randomized and CST-controlled EAGLE-trial. Controls consisted of well-characterized, tPA-eligible patients, avoiding a bias inherent to other historic controls. Identical criteria were used for blinded outcome assessment in our cohort and the EAGLE-trial [15].

Mean baseline BCVA observed in our IVT-cohort was significantly worse than in the EAGLE CST-arm. This may have led to a bias in favor of IVT effect expressed by the overall gain in mean LogMAR from baseline to d5/d30, where mean BCVA values did not differ significantly between groups. However, despite worse baseline BCVA, more patients experienced functional recovery, which supports our assumption of a real IVT effect.

We cannot rule out a bias in favor of our IVT-cohort caused by transient NA-CRAO with spontaneous recovery [3]. Mean onset to treatment delay in the EAGLE CST-arm was eleven hours versus three hours in our IVT-cohort, which makes inclusion of transient NA-CRAO more likely in our cohort.

The EAGLE-trial did not address the cause of NA-CRAO; thus, direct comparison to our IVT-cohort is limited since clots’ origin might significantly influence IVT response and visual outcomes.

No fluorescein angiography was performed prior to treatment in our trial. Consequently, there remains a risk of non-CRAO inclusions. However, even guidelines recommend skipping fluorescein angiography in the acute phase in favor of an undelayed hospital admission for immediate treatment and neurovascular work-up [2].

For the same reasons, no initial assessment of the visual field was performed. This may be crucial for distinguishing true recovery of retinal function and apparent recovery, which occurs through adaption to central visual loss by eccentric fixation [3]. However, a BCVA of LogMAR ≤0.5 Snellen equivalent: 6/20) may not be achieved through eccentric fixation as spatial resolution in the peripheral visual field (>2° from the center of foveal vision) is insufficient [22].

Finally, we did not perform retrobulbar ultrasound prior or parallel to IVT in our study. Thus, we can neither make assumptions on the embolus’ composition nor the spot sign’s prognostic value regarding IVT efficacy [11].

## Conclusions

The clinical data collected in this case series suggests potential efficacy and safety of IVT as a treatment for acute NA-CRAO if initiated within 4.5 hours after symptom onset. Our outcome data reveals relevant levels of disability in patients who do not recover from visual loss due to NA-CRAO. This instance may outweigh the potential risks of thrombolytic treatment.

A prospective, randomized, placebo-controlled phase III trial, as proposed above, is warranted to evaluate efficacy and safety of very early IVT in acute NA-CRAO.

## Supporting information

S1 Table In- and exclusion criteria for intravenous thrombolysis. (DOCX)

S2 Table Individual characteristics and outcomes of patients #1 to #10 and #11 to #20. (DOCX)
