# Supplementary material for: Intravenous thrombolysis in acute central retinal artery occlusion – A prospective interventional case series
Source: PLoS One. 2018 May 29;13(5):e0198114. doi: 10.1371/journal.pone.0198114 (PMC5973600; doi:10.1371/journal.pone.0198114)
Supplement: S1 Table — (DOCX) [file pone.0198114.s001.docx]

**S1 In- and exclusion criteria for intravenous thrombolysis**

| **Inclusion criteria** |
| --- |
| Clinical diagnosis of a central retinal artery occlusion (i.e. sudden, painless monocular visual loss) assessed by an experienced ophthalmologist including compatible fundoscopy |
| Known symptom onset <4.5 hours |
| Best corrected visual acuity of LogMAR ≥1.3 (Snellen equivalent: 6/120) |
| Neurological examination performed by an experienced stroke neurologist |
| Exclusion of intracranial hemorrhage by computed tomography or magnetic resonance imaging |
| Age ≥18 years |
| Written informed consent |
| **Exclusion criteria** |
| Best corrected visual acuity of LogMAR <1.3 (Snellen equivalent: 6/120) or rapidly improving vision |
| Intracranial hemorrhage on computed tomography or magnetic resonance imaging |
| Clinical presentation suggests subarachnoid hemorrhage |
| Neurosurgery, head trauma, or stroke in past 3 months |
| Uncontrolled hypertension (>185 mmHg systolic blood pressure or >110 mmHg diastolic blood pressure) |
| History of intracranial hemorrhage |
| Known intracranial arteriovenous malformation, neoplasm, or aneurysm |
| Active internal bleeding |
| Suspected/confirmed endocarditis |
| Known bleeding diathesis* |
| Abnormal blood glucose (<50 or >400 mg/dL) |
| Major surgery or serious non-head trauma in the previous 14 days |
| History of gastrointestinal or urinary tract hemorrhage within 21 days |
| Recent arterial puncture at a non-compressible site |
| Recent lumbar puncture |
| Post myocardial infarction pericarditis |
| Pregnancy |

*Known bleeding diathesis comprises (1) patient has received heparin within 48 hours, (2) current use of vitamin K antagonists (e.g. warfarin or phenprocoumon) and an elevated INR >1.7 (assessed via point-of-care coagulation testing), and (3) the use of direct thrombin inhibitors (e.g. dabigatran) or direct factor Xa inhibitors (e.g. apixaban, edoxaban or rivaroxaban) within past 48 hours. As per standard operating procedure and in order to avoid treatment delay, thrombolysis is started before lab-results become available; in case of platelet count <100,000 per µL or an elevated activated partial thromboplastin time thrombolysis is stopped immediately.
