# Supplementary material for: Intravenous thrombolysis in acute central retinal artery occlusion – A prospective interventional case series
Source: PLoS One. 2018 May 29;13(5):e0198114. doi: 10.1371/journal.pone.0198114 (PMC5973600; doi:10.1371/journal.pone.0198114)
Supplement: S2 Table — (DOCX) [file pone.0198114.s002.docx]

**S2 Table. Individual characteristics and outcomes of patients #1 to #10 and #11 to #20**

| **Patients #1 to #10** | **#1** | **#2** | **#3** | **#4** | **#5** | **#6** | **#7** | **#8** | **#9** | **#10** |
| --- | --- | --- | --- | --- | --- | --- | --- | --- | --- | --- |
| Sex, female | 1 | 0 | 1 | 0 | 0 | 0 | 1 | 1 | 0 | 0 |
| Age, years | 80 | 74 | 85 | 65 | 47 | 84 | 70 | 64 | 60 | 64 |
| Body weight, kg | 56 | 90 | 70 | 105 | 115 | 75 | 90 | 50 | 86 | 80 |
| **Vascular risk factors** |  |  |  |  |  |  |  |  |  |  |
| Arterial hypertension | 1 | 1 | 1 | 1 | 1 | 1 | 1 | 0 | 1 | 1 |
| Diabetes mellitus | 0 | 1 | 0 | 1 | 0 | 1 | 0 | 0 | 0 | 1 |
| Hyperlipidemia | 1 | 1 | 1 | 1 | 1 | 0 | 1 | 1 | 1 | 1 |
| Atrial fibrillation | 0 | 0 | 0 | 0 | 0 | 0 | 0 | 0 | 0 | 0 |
| Ischemic heart disease or history of myocardial infarction | 0 | 1 | 1 | 0 | 0 | 0 | 0 | 0 | 0 | 0 |
| Congestive heart failure | 0 | 1 | 0 | 0 | 0 | 0 | 0 | 0 | 0 | 0 |
| Active smoking | 0 | 1 | 0 | 0 | 1 | 1 | 0 | 1 | 1 | 0 |
| History of stroke | 0 | 0 | 0 | 0 | 0 | 1 | 0 | 0 | 0 | 0 |
| **Prior antithrombotic treatment** | 0 | ASA | ASA | 0 | 0 | ASA | 0 | 0 | 0 | 0 |
| **Ophthalmological assessment** |  |  |  |  |  |  |  |  |  |  |
| Affected eye, right | 0 | 0 | 1 | 1 | 1 | 0 | 0 | 1 | 0 | 1 |
| Historical BCVA of affected eye, LogMAR | 0 | 0 | 0 | 0 | 0 | 0 | 0 | 0 | 0 | 0 |
| BCVA prior to IVT, LogMAR | 2,6 | 2 | 2,6 | 2,6 | 2,6 | 2,9 | 2,6 | 2,6 | 2,6 | 2,3 |
| Conservative standard treatment prior to IVT | 0 | 0 | 0 | 0 | 0 | 0 | 0 | 0 | 0 | 0 |
| Symptom onset to initiation of IVT, minutes | 210 | 180 | 180 | 90 | 225 | 140 | 210 | 120 | 240 | 240 |
| BCVA at day 5 ±2 (or discharge), LogMAR | 1,3 | 0,2 | 2,3 | 0,1 | 1,3 | 2,9 | 2,3 | 0,2 | 2,9 | 2,6 |
| BCVA at day 30 ±5, LogMAR | 1,3 | 0 | 2,3 | 1,5 | 0,8 | 2,9 | 2,3 | 0,2 | 2,9 | 2,6 |
| BCVA at latest follow-up, LogMAR | 0,4 | 0 | 2,3 | 1,5 | 0,4 | n/a | n/a | 0,2 | 2,9 | 2,6 |
| Time from NA-CRAO to last follow-up, months | 10 | 9 | 6 | 12 | 18 | n/a | n/a | 6 | 30 | 5 |
| **Neurological assessment** |  |  |  |  |  |  |  |  |  |  |
| Premorbid modified Rankin Scale score | 0 | 1 | 0 | 0 | 0 | 3 | 0 | 0 | 0 | 0 |
| NIHSS prior to IVT | 0 | 0 | 0 | 0 | 0 | 1 (LOC questions, due to dementia) | 0 | 0 | 0 | 0 |
| Systolic blood pressure prior to IVT, mmHg | 152 | 156 | 165 | 155 | 161 | 134 | 148 | 162 | 151 | 152 |
| NIHSS at day 5 ±2 (or discharge) | 0 | 0 | 0 | 0 | 0 | 0 | 0 | 0 | 0 | 0 |
| modified Rankin Scale score at day 30 ±5 | 1 | 1 | 2 | 2 | 2 | 3 | 2 | 1 | 2 | 2 |
| **Neuroimaging** |  |  |  |  |  |  |  |  |  |  |
| CT or MRI prior to IVT | CT | CT | CT | CT | CT | CT | CT | CT | CT | CT |
| Old cerebral infarcts | 0 | 0 | 0 | 1 | 0 | 1 | 0 | 0 | 0 | 0 |
| White matter changes (0 none, + mild, ++ moderate) | ++ | ++ | ++ | + | 0 | + | + | + | + | + |
| CT or MRI at 24 ±12 hours | CT | CT | CT | MRI | CT | CT | CT | CT | CT | MRI |
| New cerebral infarcts | 0 | 0 | 0 | 1 | 0 | 0 | 0 | 0 | 0 | 0 |
| **Adverse Events** |  |  |  |  |  |  |  |  |  |  |
| Serious adverse events | 0 | 0 | 0 | 2 (orolingual angioedema, recurrent NA-CRAO) | 0 | 1 (abdominal aortic aneurysm bleeding) | 0 | 0 | 0 | 0 |
| Any bleeding complications | 0 | 0 | 0 | 0 | 0 | 1 | 0 | 0 | 0 | 0 |
| **Etiology of NA-CRAO** |  |  |  |  |  |  |  |  |  |  |
| Suspected NA-CRAO etiology, according to TOAST [1] classification | 1 | 1 | 5 | 5 | 5 | 5 | 5 | 5 | 5 | 5 |
| Large artery arteriosclerosis, according to [2] (i.e. severe atherosclerosis >4 mm of carotid arteries or aortic arch) | 1 | 1 | 1 | 0 | 0 | 1 | 0 | 0 | 1 | 1 |
| **Antithrombotic treatment at discharge** | DAP | DAP | CLOP | ASA | apixaban | ASA | ASA | ASA | ASA | rivaroxaban |
|  |  |  |  |  |  |  |  |  |  |  |
| **Patients #11 to #20** | **#11** | **#12** | **#13** | **#14** | **#15** | **#16** | **#17** | **#18** | **#19** | **#20** |
| Sex, female | 1 | 1 | 0 | 1 | 1 | 0 | 1 | 0 | 1 | 0 |
| Age, years | 82 | 92 | 64 | 79 | 85 | 75 | 75 | 75 | 61 | 75 |
| Body weight, kg | 80 | 55 | 70 | 75 | 60 | 101 | 75 | 75 | 55 | 55 |
| **Vascular risk factors** |  |  |  |  |  |  |  |  |  |  |
| Arterial hypertension | 1 | 1 | 0 | 1 | 1 | 1 | 1 | 1 | 0 | 1 |
| Diabetes mellitus | 1 | 0 | 0 | 0 | 0 | 0 | 1 | 0 | 0 | 0 |
| Hyperlipidemia | 1 | 0 | 0 | 0 | 1 | 1 | 0 | 1 | 1 | 0 |
| Atrial fibrillation | 0 | 0 | 0 | 1 | 0 | 0 | 0 | 0 | 0 | 0 |
| Ischemic heart disease or history of myocardial infarction | 1 | 0 | 0 | 0 | 0 | 0 | 1 | 0 | 0 | 0 |
| Congestive heart failure | 1 | 0 | 0 | 0 | 1 | 0 | 0 | 0 | 0 | 0 |
| Active smoking | 0 | 0 | 0 | 0 | 0 | 0 | 0 | 1 | 0 | 1 |
| History of stroke | 0 | 0 | 0 | 1 | 1 | 0 | 0 | 1 | 0 | 0 |
| **Prior antithrombotic treatment** | ASA | ASA | 0 | 0 | 0 | ASA | DAP | CLOP | 0 | 0 |
| **Ophthalmological assessment** |  |  |  |  |  |  |  |  |  |  |
| Affected eye, right | 1 | 1 | 0 | 1 | 0 | 1 | 1 | 1 | 1 | 1 |
| Historical BCVA of affected eye, LogMAR | 0 | 1 | 0 | 0 | 0 | 0 | 0 | 0 | 0 | 0 |
| BCVA prior to IVT, LogMAR | 2,3 | 2,3 | 2,6 | 2,9 | 2,9 | 2 | 2,3 | 2,6 | 1,6 | 2,3 |
| Conservative standard treatment prior to IVT | 0 | brimonidine eye drops | 0 | 0 | 0 | dorzolamide /timolol eye drops | 0 | 0 | 0 | 0 |
| Symptom onset to initiation of IVT, minutes | 210 | 240 | 210 | 120 | 90 | 105 | 240 | 260 | 270 | 90 |
| BCVA at day 5 ±2 (or discharge), LogMAR | 2,3 | 1 | 2 | 2,3 | 0,1 | 1,3 | 2,6 | 2,6 | 0 | 0 |
| BCVA at day 30 ±5, LogMAR | 2,3 | 1 | 2 | 2,3 | 0,1 | 2,3 | 2,6 | 2,6 | 0 | 0 |
| BCVA at latest follow-up, LogMAR | 2,3 | n/a | n/a | 2,3 | n/a | 2,3 | 2,3 | n/a | 0 | 0 |
| Time from NA-CRAO to last follow-up, months | 22 | n/a | n/a | 4 | n/a | 2 | 2 | n/a | 2 | 4 |
| **Neurological assessment** |  |  |  |  |  |  |  |  |  |  |
| Premorbid modified Rankin Scale score | 2 | 3 | 0 | 0 | 3 | 0 | 1 | 0 | 0 | 0 |
| NIHSS prior to IVT | 0 | 1 (mild sensory loss of right face and arm) | 0 | 0 | 0 | 0 | 0 | 0 | 0 | 0 |
| Systolic blood pressure prior to IVT, mmHg | 158 | 165 | 131 | 166 | 141 | 166 | 135 | 174 | 133 | 150 |
| NIHSS at day 5 ±2 (or discharge) | 0 | 0 | 0 | 0 | 0 | 0 | 0 | 0 | 0 | 0 |
| modified Rankin Scale score at day 30 ±5 | 2 | 3 | 2 | 2 | 3 | 2 | 2 | 2 | 0 | 0 |
| **Neuroimaging** |  |  |  |  |  |  |  |  |  |  |
| CT or MRI prior to IVT | MRI | CT | CT | CT | CT | CT | CT | CT | CT | CT |
| Old cerebral infarcts | 0 | 0 | 0 | 1 | 1 | 0 | 0 | 1 | 0 | 0 |
| White matter changes (0 none, + mild, ++ moderate) | + | ++ | 0 | 0 | ++ | ++ | + | 0 | 0 | 0 |
| CT or MRI at 24 ±12 hours | MRI | CT | MRI | MRI | CT | CT | MRI | CT | CT | CT |
| New cerebral infarcts | 1 | 0 | 1 | 0 | 0 | 0 | 0 | 0 | 0 | 0 |
| **Adverse Events** |  |  |  |  |  |  |  |  |  |  |
| Serious adverse events | 0 | 0 | 0 | 0 | 0 | 0 | 0 | 0 | 0 | 0 |
| Any bleeding complications | 0 | 0 | 0 | 0 | 0 | 0 | 0 | 0 | 0 | 0 |
| **Etiology of NA-CRAO** |  |  |  |  |  |  |  |  |  |  |
| Suspected NA-CRAO etiology, according to TOAST [1] classification | 5 | 5 | 5 | 2 | 5 | 5 | 5 | 1 | 5 | 5 |
| Large artery arteriosclerosis, according to [2] (i.e. severe atherosclerosis >4 mm of carotid arteries or aortic arch) | 1 | 0 | 0 | 1 | 1 | 1 | 0 | 1 | 0 | 1 |
| **Antithrombotic treatment at discharge** | DAP | CLOP | ASA | dabigatran | ASA | ASA | DAP | CLOP | ASA | ASA |

ASA = acetylsalicylic acid, BCVA = best corrected visual acuity, CLOP = clopidogrel, CT = computed tomography, DAP = dual antiplatelet therapy, IVT = intravenous thrombolysis, LOC = level of consciousness, LogMAR = logarithm of the minimum angle of resolution, MRI = magnetic resonance imaging, n/a = not applicable, NA-CRAO = non-arteritic central retinal artery occlusion, NIHSS = National Institutes of Health Stroke Scale score, TOAST = Trial of Org 10172 in Acute Stroke Treatment [1] (1: large-artery atherosclerosis, 2: cardioembolism, 3: small-vessel occlusion, 4: stroke of other determined etiology, and 5: stroke of undetermined etiology).

**SUPPLEMENTAL REFERENCES**

1. Adams HP, Jr., Bendixen BH, Kappelle LJ, Biller J, Love BB, Gordon DL, et al. Classification of subtype of acute ischemic stroke. Definitions for use in a multicenter clinical trial. Toast. Trial of org 10172 in acute stroke treatment. *Stroke*. 1993;24:35-41

2. Nedelmann M, Graef M, Weinand F, Wassill KH, Kaps M, Lorenz B, et al. Retrobulbar spot sign predicts thrombolytic treatment effects and etiology in central retinal artery occlusion. *Stroke*. 2015;46:2322-2324
